# Acrolein-Triggered Ferroptosis and Protection by Intermittent Fasting via the AMPK/NRF2-CLOCK/BMAL1 Pathway

**DOI:** 10.3390/toxics13050369

**Published:** 2025-05-01

**Authors:** Yuandie Zhang, Hong Chen, Qianfeng Chen, Margaret Zaitoun, Ying Cheng, Jierong Ge, Qing Feng

**Affiliations:** Department of Nutrition and Food Hygiene, Key Laboratory of Toxicology, School of Public Health, Key Laboratory of Public Health Safety and Emergency Prevention and Control Technology of Higher Education Institutions in Jiangsu Province, Nanjing Medical University, Nanjing 211166, China; yuandie980323@163.com (Y.Z.); chenhong_00@126.com (H.C.); cqf810810@163.com (Q.C.); margaret88@stu.njmu.edu.cn (M.Z.); yingcheng1013@163.com (Y.C.); gejierong@foxmail.com (J.G.)

**Keywords:** acrolein, intermittent fasting, ferroptosis, circadian clock, AMPK, NRF2

## Abstract

Environmental pollution significantly exacerbates various diseases, particularly those affecting the cardiovascular and respiratory systems. Our previous studies have shown that acrolein, an environmental pollutant, promotes atherosclerosis by downregulating the circadian clock genes (CLOCK/BMAL1) and disrupting circadian rhythm. We have also found that intermittent fasting (IF), closely linked to the circadian clock, may mitigate atherosclerosis induced by acrolein. Ferroptosis, a newly identified form of regulated cell death, is associated with the acceleration of atherosclerotic development, but its relationship with the circadian clock is not well understood. In this study, we explored the potential of IF to alleviate ferroptosis by modulating the circadian clock. Our in vivo experiments revealed that IF reversed ferroptosis and upregulated CLOCK/BMAL1 in APOE-/- mice. In human umbilical vein endothelial cells (HUVECs), we discovered that acrolein-induced ferroptosis leads to cell death, while short-term starvation (STS, IF cell model) reversed this effect. Acrolein also suppressed the expression of AMP-activated protein kinase (AMPK), nuclear factor erythroid 2-related factor 2 (NRF2), and CLOCK/BMAL1, which were restored by subsequent STS treatments. Additionally, the overexpression of CLOCK/BMAL1 mitigated ferroptosis, consistent with findings from CLOCK gene knockout experiments. Notably, CLOCK/BMAL1 and AMPK/NRF2 were found to be mutually regulated. Concurrently, the AMPK and NRF2 signaling pathways may be interdependent and act in concert. In conclusion, our findings suggest that IF modulates the CLOCK/BMAL1-AMPK/NRF2 pathway to alleviate acrolein-induced ferroptosis, offering a potential strategy to address health issues related to environmental pollution.

## 1. Introduction

Environmental pollution significantly impacts human health [1]. Acrolein is an α, β-unsaturated aldehyde commonly found in cigarette smoke, automobile exhaust, cooking fumes, and certain foods [2,3]. Acrolein has been implicated in the pathogenesis of several diseases, including multiple sclerosis, neurodegenerative disorders such as Alzheimer’s disease, cardiovascular and respiratory diseases, diabetes mellitus, and cancer [4]. Furthermore, acrolein serves as a crucial intermediate in the chemical industry for the synthesis of various organic compounds and in the production of methionine and chloromethane refrigerants [5,6]. Thus, the general population is exposed to acrolein through a wide range of avenues [7].

Our initial studies have shown that multiple dietary factors could antagonize the adverse effects of acrolein. First of all, olive leaf extract could prevent the negative effects of acrolein on the myocardium and cardiomyocytes [8]. In addition, asparagus extract can protect smooth muscle cells from acrolein-induced apoptosis by regulating the circadian clock [9]. Moreover, the circadian clock and gut microbiota might be promising targets for the prevention of cardiovascular disease caused by acrolein [10]. Our antecedent study has also established that intermittent fasting (IF) improves acrolein-induced atherosclerosis by regulating the expression of CLOCK/BMAL1 [11]. These results suggest that the circadian clock plays a role in the regulation of acrolein-induced adverse effects, and there is a significant correlation between the circadian clock and IF.

IF is a dietary intervention intimately linked to the circadian clock [12]. IF encompasses various strategies, including time-restricted feeding (TRF), alternate-day fasting (ADF), and modified fasting (MF) regimens [13]. This approach has significant implications for health, aging, and disease management, establishing itself as an effective intervention for the prevention and treatment of diverse medical conditions [14]. As a dietary pattern, IF is not a direct restriction of energy intake but a fasting during a specific time and a free diet at other times, which is safer than the energy restriction [15]. It has been demonstrated that IF can reduce risk factors associated with atherosclerosis, like inflammatory factors and blood lipid levels [16]. Furthermore, in the study of IF, “STS” refers to “Short-Term Starvation”. This model is used in IF research to elucidate the response and adaptation mechanisms of cells subjected to short-term starvation [17]. Human umbilical vein endothelial cells (HUVECs) are frequently utilized in IF studies to investigate mechanisms related to endothelial function, metabolic regulation, and cardiovascular health, offering a valuable model for comprehending the effects of IF on cellular and overall health. Consequently, in this study, we employed HUVECs to construct the STS model.

The circadian clock is a regulatory system that governs biological rhythm and numerous physiological processes in response to the 24-hour light-dark cycle [18]. The mammalian circadian clock system comprises a central circadian clock located in the suprachiasmatic nucleus of the hypothalamus, as well as peripheral circadian clocks found in the liver, kidneys, intestines, and other organs [19]. At the cellular level, the circadian clock is governed by a negative transcription–translation feedback loop involving core genes, such as CLOCK and BMAL1, which drive molecular oscillations and establish rhythmic cellular behavior [20,21]. In addition to this, the circadian clock directly regulates various cardiovascular functions, including endothelial function, thrombosis, blood pressure, heart rate, and vascular endothelial integrity [22]. Disturbances in circadian rhythms are thought to contribute to the development of cardiovascular disease [23]. Consequently, prolonged exposure to shift work, night shifts, jet lag, and sleep disorders significantly elevates the risk of developing cardiovascular disease [24,25].

Scientific evidence has demonstrated that IF can reduce both lipid peroxidation and mitochondrial dysfunction, thereby alleviating the characteristic cellular and mouse damage induced by ferroptosis [26]. Ferroptosis is an iron-dependent, oxidative form of non-apoptotic regulatory cell death, characterized by an increase in free iron and the accumulation of lipid peroxides [27]. This process is marked by a reduction in the activity of glutathione peroxidase 4 (GPX4) [28], along with an increase in the expression of the transferrin receptor TFR1 (TFRC) [29] and acyl-CoA synthase (ACSL4) [30]. Furthermore, ferroptosis has been implicated in the development of various pathological conditions, including cancer [31], neurodegenerative diseases [32], and atherosclerosis [33]. The evidence indicates that acrolein is capable of inducing ferroptosis [34]. While our previous studies have demonstrated that IF improves acrolein-induced atherosclerosis by regulating the expression of CLOCK/BMAL1 [11], the relationship between ferroptosis and the circadian clock, as well as the underlying mechanisms involved, remains to be elucidated. Given the intricate interplay among circadian rhythm, cardiovascular disease, and ferroptosis elucidated in the preceding discourse, we have selected TRF as the in vivo model for our experimental investigation.

IF is also closely associated with energy-responsive molecules [35], such as AMP-activated protein kinase (AMPK). As a key energy regulator, AMPK plays a significant role in the pathophysiology of disorders including diabetes, cardiometabolic disease, and cancer [36]. Moreover, AMPK acts as a sensor of cellular energy status; its inactivation substantially abolishes the protective effects of energy stress against ferroptosis in vitro, as well as ferroptosis-related renal ischemia–reperfusion injury in vivo. Notably, cancer cells exhibiting high basal AMPK activation demonstrate resistance to ferroptosis, whereas AMPK inactivation sensitizes these cells to ferroptosis [14]. Furthermore, NRF2, or nuclear factor erythroid 2-related factor 2, functions as a transcription factor involved in the cellular stress response and various physiological processes [37]. Owing to its capacity to target numerous genes implicated in the ferroptosis cascade, NRF2 has been identified as a crucial regulator of ferroptosis, playing a pivotal role in this mechanism. It is noteworthy that the observed overlapping cellular responses upon activation of AMPK or NRF2, as well as the common stressors that impinge on both AMPK and NRF2 signaling pathways, provide a basis for the hypothesis that these two signaling pathways may be interdependent and act in concert to restore cellular homeostasis [38]. Therefore, the activation of the AMPK/NRF2 pathway can effectively modulate ferroptosis [39,40]. Our previous study indicates that IF improves acrolein-induced atherosclerosis by regulating AMPK [11]; thus, this study examines the interplay between AMPK and NRF2 to delineate the link between IF and ferroptosis.

As previously discussed, it remains uncertain whether IF suppresses ferroptosis through the circadian clock. Therefore, we aimed to investigate the effects of IF on acrolein-induced ferroptosis through the circadian clock and to elucidate its potential molecular mechanisms. We anticipate that this study will offer a novel perspective for the prevention and treatment of diseases induced by environmental pollution.

## 2. Materials and Methods

### 2.1. Cell Culture and Reagents

The HUVECs were cultured in DMEM (YIFEIXUE BIOTECH Co., Ltd., Nanjing, China) supplemented with 10% fetal bovine serum (Gibco, Grand Island, NY, USA) and 1% penicillin–streptomycin solution (Beyotime, Shanghai, China). The cells were incubated at 37 °C in a 5% CO_2_ atmosphere. We divided the cells into four groups: control, acrolein, STS, and acrolein + STS. Once the cells reached optimal growth conditions, the control group was left untreated, and the acrolein group was treated with the DMEM medium containing acrolein at a concentration of 20 μM for 24 h. The STS group was treated with STS medium (composed of 50 mL of sugar-free DMEM medium supplemented with 0.5 mL of serum and anhydrous glucose at a concentration of 0.25 mg) for 12 h. Lastly, the acrolein + STS group received DMEM medium with acrolein for 12 h, followed by a treatment with STS medium containing acrolein at a concentration of 20 μM for another 12 h.

### 2.2. Western Blot Analysis

The proteins were extracted from cells or mice tissues using cold protein lysis buffer (RIPA, Beyotime, Shanghai, China) supplemented with PMSF (protease and phosphatase inhibitors). Subsequently, the samples were separated by SDS/PAGE (sodium dodecyl sulfate polyacrylamide gel electrophoresis; Invitrogen, Carlsbad, USA) and transferred onto nitrocellulose (NC) membranes. Following blocking with skim milk, the membranes were incubated overnight with primary antibodies and then for 1 h with secondary antibodies. The specific antibodies used in this study included CLOCK, BMAL1, ACSL4, GPX4, TFRC, and GAPDH (Rosemont, IL, USA). A semi-quantitative analysis of protein bands was performed using the ImageJ version 1.52p (National Institutes of Health, Bethesda, MD, USA), while GAPDH served as the loading control.

### 2.3. RNA Isolation and Real-Time Quantitative PCR (RT-qPCR)

Total RNA was extracted from cells using RNAiso Plus (YIFEIXUE BIOTECH, Nanjing, China) and reverse transcribed into cDNA with a Prime Script TM RT premixed solution (TaKaRaBio Technology, Dalian, China). RNA quantification was performed by qPCR using SYBR Premix Ex Taq II (TaKaRaBio Technology, Dalian, China), and the primers used are listed in Table 1 and Table 2. All PCR analyses were conducted following either the 2-∆∆CT or 2-∆CT method.

### 2.4. Malondialdehyde (MDA) Assay

The HUVECs were enumerated and seeded onto a 6-well plate overnight at a density of 2 × 10^5^ cells/well; then, we divided the cells into three groups: control, acrolein, and acrolein + STS. Lipid peroxidation was assessed using the MDA detection kit (S0131S; Beyotime Biotechnology, Shanghai, China). After determining the protein concentration, the malondialdehyde levels were quantified through colorimetry according to the manufacturer’s protocol. The absorbance measurements were taken at 523 nm.

### 2.5. siRNA and Plasmid Transfection

A small interfering RNA (siRNA)-targeting CLOCK was designed and purchased from Ribobio (Guangzhou, China). The CLOCK/BMAL1 plasmids were designed and purchased from Corues Biotechnology (Nanjing, China). The HUVECs were cultured in 6-well plates at a density of 1 × 10^5^ cells/well until they adhered to the wall at 37 °C. Then, the medium was changed to DMEM without penicillin and cultured overnight until the cell density reached 70%~80%. Lipofectamine 2000 reagent (Invitrogen) was used for the siRNA and plasmid transfection according to the following procedure: (1) mix 200 μL of opti-MEM with 10 μL of Lipo2000; (2) mix 200 μL of opti-MEM with either 10 μL of siRNA or plasmid; (3) after 5 min, combine the step-one and step-two liquids, and let them stand for twenty minutes; (4) wash the cells twice with PBS before adding a mixture of 1600 μL of opti-MEM and 400 μL of the mixture into each well; (5) after transfection for 4 to 6 h, change the cells into a normal medium for further culture or collection.

### 2.6. In Vivo Studies

A total of 16 APOE-/- mice (6–7 weeks, 20–25 g) were purchased from the Model Animal Research Center of Nanjing University (Nanjing, Jiangsu, China) and raised in the Laboratory Animal Center of Nanjing Medical University. The mice were randomly divided into 4 groups, with 4 mice in each group. The detailed grouping process is as follows: control group, free feeding without acrolein; TRF group, free feeding for 6 h (18:00–24:00) every day and fasting in other periods; acrolein group, 3 mg/kg·d acrolein was added through drinking water; acrolein + TRF group, free feeding for 6 h (18:00–24:00) on the basis of adding 3 mg/kg acrolein to daily drinking water. The water intake and body weight of the mice were measured once a week to adjust the amount of acrolein in their drinking water. After 14 weeks, the mice were euthanized, and their liver and heart tissues were collected for further analysis.

### 2.7. Statistical Analysis

All data were reported as an average ± SD. The SPSS 25.0 software (SPSS Inc. IBM, Chicago, IL, USA) and GraphPad Prism 8.0 (GraphPad Prism, Inc, La Jolla, CA, USA) were used for the statistical analyses. We considered the comparisons between the quantitative variables statistically significant if *p* < 0.05, as determined using a student’s *t*-test and one-way ANOVA, followed by the Bonferroni multiple comparison test.

## 3. Results

### 3.1. IF Attenuates Acrolein-Induced Ferroptosis and Low Expression of CLOCK In Vivo

**Figure 1:**
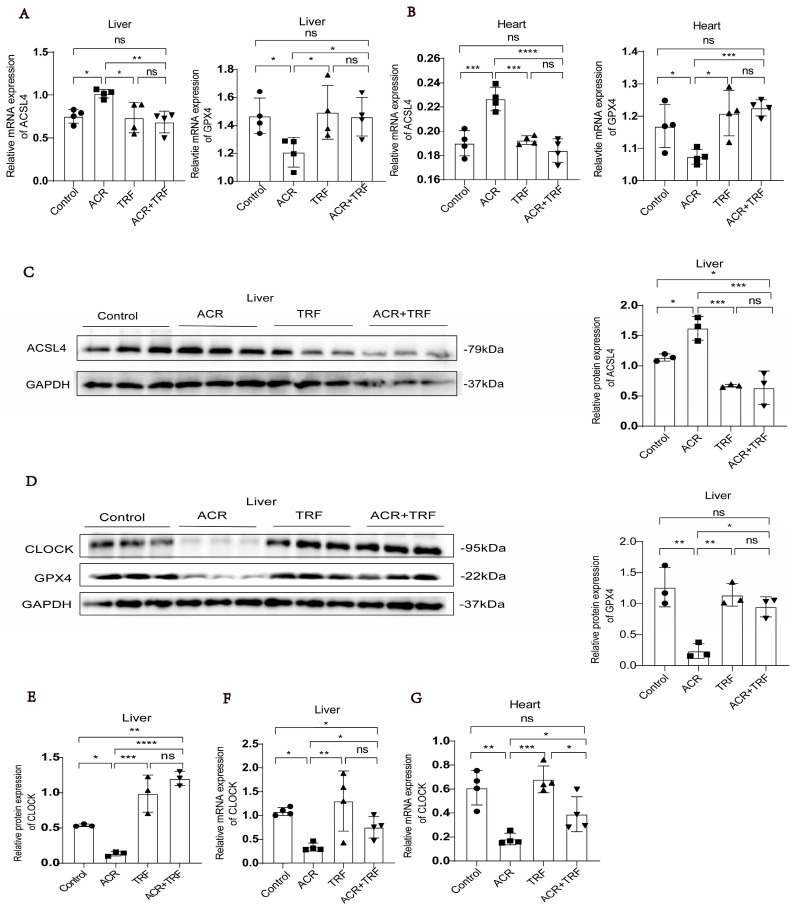
Expression of ACSL4/GPX4/CLOCK following treatment with acrolein and/or TRF. (**A**) The liver mRNA expression levels of ferroptosis markers ACSL4 and GPX4 were assessed in APOE-/- mice (*n* = 4). (**B**) The heart mRNA expression levels of ACSL4 and GPX4, which serve as markers for ferroptosis, were measured in APOE-/- mice (*n* = 4). (**C**–**E**) The protein expression levels of ACSL4, GPX4, and CLOCK were assessed in the liver tissue of APOE-/- mice (*n* = 3). (**F**,**G**) The mRNA expression levels of CLOCK were examined in the liver and heart tissue of APOE-/- mice (*n* = 4). Data are presented as mean ± standard deviation from at least three independent experiments and analyzed with one-way ANOVA, followed by the Bonferroni multiple comparison test. ns: no significance, * *p* < 0.05, ** *p* < 0.01, *** *p* < 0.001, and **** *p* < 0.0001.

Our prior study has shown that IF improves acrolein-induced atherosclerosis by regulating the expression of CLOCK/BMAL1 [11]. To further explore the mechanism of IF on ferroptosis triggered by acrolein, we divided mice into four groups: control, acrolein, TRF, and acrolein + TRF. We found that the expression of GPX4 was higher in the TRF and acrolein + TRF groups compared to that in the acrolein group. On the contrary, the expression of ACSL4 was lower in the TRF and acrolein + TRF groups compared to that in the acrolein group (Figure 1A–D). The evidence confirms that IF is strongly associated with the circadian clock; therefore, we detected that IF can restore circadian clock gene expression levels suppressed by acrolein. We found that the expression of CLOCK was higher in the TRF and acrolein + TRF groups compared to that in the acrolein group (Figure 1D–G). Our previous study also observed that the expression of BMAL1 was elevated in the TRF and acrolein + TRF groups compared to the acrolein group in in vivo experiments [11]. Collectively, these experimental results provide evidence supporting the inhibitory effects of IF on both acrolein-induced ferroptosis and the downregulation of CLOCK/BMAL1. However, it should be noted that ferroptosis inhibitors, such as ferrostatin-1, were not utilized in our in vivo experiments. Consequently, although alterations in ferroptosis-related markers were detected, the direct functional validation of ferroptosis suppression in vivo remains to be explored in future studies.

### 3.2. Acrolein Induces Ferroptosis and Inhibits the Expression of AMPK/NRF2 and CLOCK/BMAL1 in HUVECs

**Figure 2:**
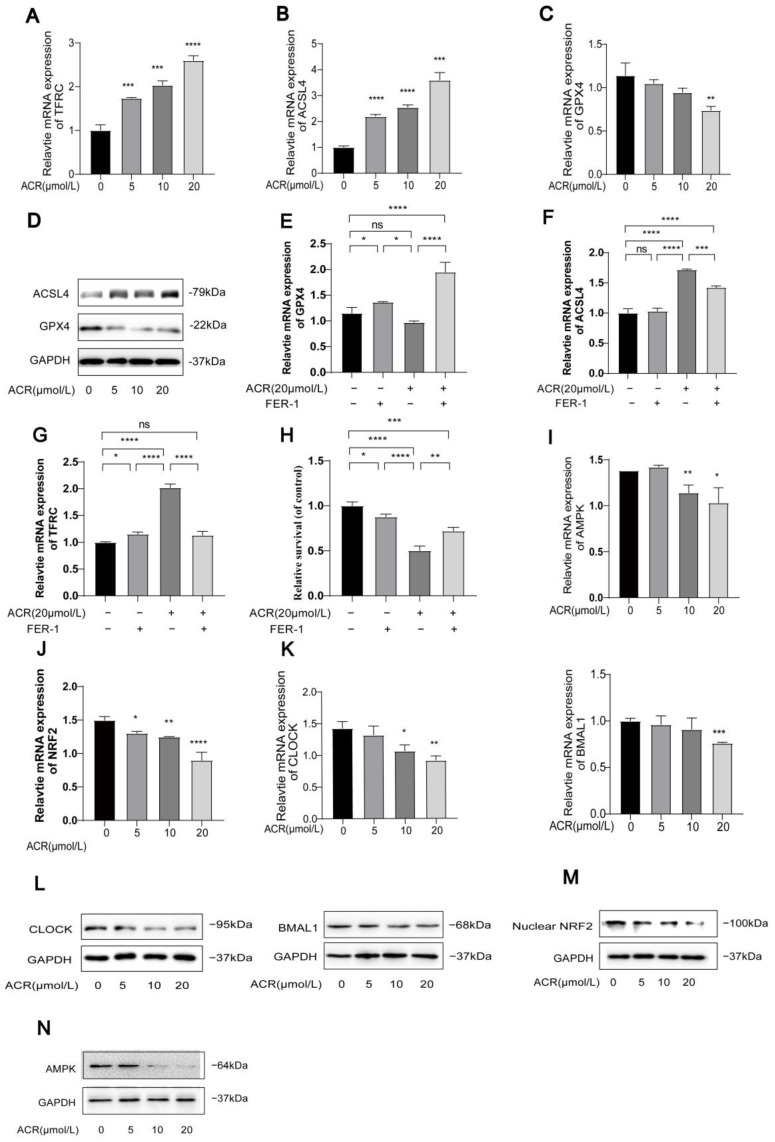
Acrolein induces the ferroptosis and the downregulation of AMPK/NRF2 and CLOCK/BMAL1 in HUVECs. (**A**–**D**) The protein and mRNA expression levels of the ferroptosis markers GPX4, ACSL4, and TFRC in HUVEC were assessed after treatment with various concentrations of acrolein. (**E**–**G**) mRNA expression levels of GPX4, ACSL4, and TFRC in HUVECs. The cells were treated with acrolein (20 µM) in the presence or absence of Fer-1 (1 µM) for 24 h. (**H**) Cell viability of HUVECs, as measured by an MTT assay, after treatment with acrolein (20 µM) in the presence or absence of Fer-1 (1 µM) for 24 h. (**I**–**K**) The impact of different concentrations of acrolein on the mRNA expression levels of AMPK/NRF2/CLOCK/BMAL1 in HUVECs was evaluated. (**L**–**N**) The impact of different concentrations of acrolein on the protein levels of AMPK/NRF2 and CLOCK/BMAL1 in HUVECs was evaluated. Data are presented as mean ± standard deviation from at least three independent experiments and analyzed with one-way ANOVA, followed by the Bonferroni multiple comparison test. ns: no significance, * *p* < 0.05, ** *p* < 0.01, *** *p* < 0.001, and **** *p* < 0.0001.

To investigate whether acrolein could induce ferroptosis and lipid peroxidation, HUVECs were treated with DMEM culture medium containing different concentrations of acrolein (0 µM, 5 µM, 10 µM, and 20 µM) for 24 h. Western blot and PCR analyses revealed that increasing the concentrations of acrolein led to elevated protein and mRNA levels of TFRC and ACSL4 (Figure 2A,B,D), while there was a decrease in protein and mRNA levels of GPX4 (Figure 2C,D). Additionally, the cells were treated with acrolein (20 µM) for 24 h, either in the presence or absence of ferrostatin-1 (Fer-1, 1 µM). The results indicated that Fer-1 reversed the acrolein-induced changes of the ferroptosis-related marker (Figure 2E–G). An MTT assay was also used to assess the cell viability, and, remarkably, we found that the treatment with Fer-1 was effective in inhibiting cell death induced by acrolein. However, it was also observed that Fer-1 could not fully restore cell viability in the MTT assay. This observation implies that mechanisms other than ferroptosis may contribute to acrolein-induced cell death, potentially attributable to the cytotoxic effects of acrolein itself. Further investigation into this aspect appears to be warranted (Figure 2H). The Western blot and PCR analyses revealed that increasing the concentrations of acrolein led to reduced protein and mRNA levels of AMPK, NRF2, CLOCK, and BMAL1 (Figure 2I–N). These findings confirm our hypothesis that acrolein can act as a promoter of ferroptosis while inhibiting the expression of AMPK/NRF2 and CLOCK/BMAL1 in HUVECs at the same time. (For all protein quantification data presented in Figure 2, please refer to Figure S1).

### 3.3. AMPK/NRF2 and CLOCK/BMAL1 Genes Are Involved in the Regulation of Ferroptosis in HUVECs 

**Figure 3:**
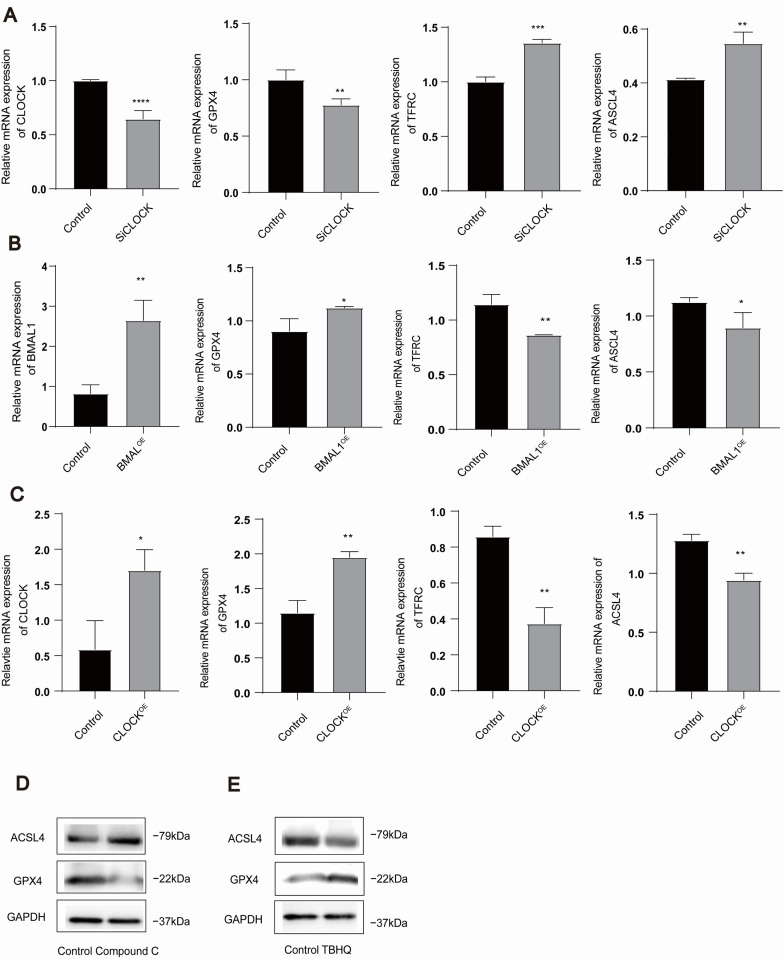
CLOCK/BMAL1 and AMPK/NRF2 regulate ferroptosis in HUVECs. (**A**) The mRNA levels of CLOCK, GPX4, TFRC, and ACSL4 were quantified using qRT-PCR following a 24-h treatment with siCLOCK in HUVECs. (**B**) The mRNA levels of BMAL1, GPX4, TFRC, and ACSL4 were measured by qRT-PCR after transfecting HUVECs with the BMAL1 plasmid for 24 h. (**C**) The mRNA levels of CLOCK, GPX4, TFRC, and ACSL4 were determined using qRT-PCR after transfection with the CLOCK plasmid in HUVECs for 24 h. (**D**) To evaluate ACSL4/GPX4 protein expression, HUVECs were treated with the AMPK inhibitor (compound C). (**E**) To evaluate ACSL4/GPX4 protein expression, HUVECs were treated with the NRF2 activator (TBHQ). Data are presented as mean ± standard deviation from at least three independent experiments and analyzed using one-way ANOVA followed by the Bonferroni multiple comparison test. ns: not significant; * *p* < 0.05; ** *p* < 0.01; *** *p* < 0.001; **** *p* < 0.0001.

We further investigated whether the AMPK/NRF2 and CLOCK/BMAL1 genes regulate the occurrence of ferroptosis in HUVECs. Our observations revealed that CLOCK knockdown downregulated GPX4 while simultaneously upregulating ACSL4 and TFRC expression (Figure 3A) (The sequences of siCLOCK are detailed in Table 3). Conversely, the overexpression of CLOCK/BMAL1 genes led to the upregulation of GPX4 and the downregulation of ACSL4 and TFRC (Figure 3B,C). Meanwhile, ompound C significantly upregulated the expression of ACSL4 while downregulating the expression of GPX4 (Figure 3D). In contrast, TBHQ downregulated the expression of ACSL4 and upregulated the expression of GPX4 (Figure 3E). These findings strongly suggest the involvement of the AMPK/NRF2 and CLOCK/BMAL1 genes in regulating ferroptosis in HUVECs. (For all protein quantification data presented in Figure 3, please refer to Figure S2).

### 3.4. Mutual Regulation Exists Between AMPK/NRF2 and CLOCK/BMAL1 in HUVECs

**Figure 4:**
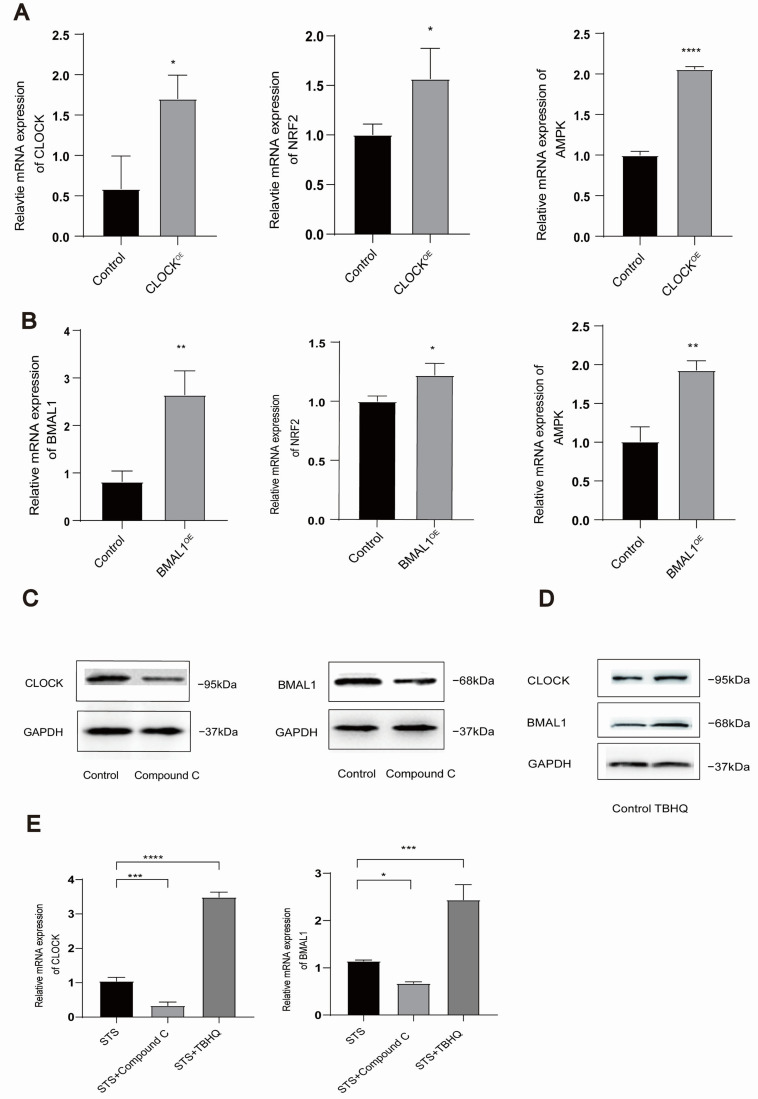
CLOCK/BMAL1/AMPK/NRF2 expression and mutual regulation in HUVECs. (**A**) The mRNA levels of AMPK/NRF2 were detected by qRT-PCR when HUVECs were transfected with CLOCK plasmid and cultured for 24 h. (**B**) The mRNA levels of AMPK/NRF2 were detected by qRT-PCR when HUVECs were transfected with BMAL1 plasmid and cultured for 24 h. (**C**) To evaluate CLOCK/BMAL1 protein expression, HUVECs were treated with the AMPK inhibitor (compound C). (**D**) To evaluate CLOCK/BMAL1 protein expression, HUVECs were treated with the NRF2 activator (TBHQ). (**E**) To evaluate CLOCK/BMAL1 mRNA expression, HUVECs were treated with STS, STS + compound C, and STS + TBHQ. Data were expressed as mean ± standard deviation of at least three experiments and analyzed by one-way ANOVA, followed by the Bonferroni multiple comparison test. ns: no significance, * *p* < 0.05, ** *p* < 0.01, *** *p* < 0.001, and **** *p* < 0.0001.

AMPK/NRF2 and the circadian clock exhibit a close relationship. Therefore, we performed overexpression of the CLOCK/BMAL1 genes in HUVECs and subsequently treated the cells with an AMPK inhibitor (compound C). The results are depicted in Figure 4. When the CLOCK/BMAL1 genes were overexpressed, the expression levels of AMPK/NRF2 mirrored the corresponding changes (Figure 4A,B). Conversely, treatment with the AMPK inhibitor resulted in decreased expression of CLOCK/BMAL1 genes (Figure 4C). Moreover, treatment with the NRF2 activator (tert-butylhydroquinone, TBHQ) led to the reduced expression of CLOCK/BMAL1 genes (Figure 4D). In addition, we investigated whether compound C and TBHQ could regulate the expression of CLOCK and BMAL1 in STS-treated cells. The results showed that compound C downregulated the expression of CLOCK and BMAL1 under fasting conditions, whereas TBHQ upregulated the expression of CLOCK and BMAL1 under the same conditions (Figure 4E). Collectively, these findings indicate a mutual regulation between the AMPK/NRF2 pathway and CLOCK/BMAL1 genes, demonstrating that their expression trends align accordingly. (For all protein quantification data presented in Figure 4, please refer to Figure S3).

### 3.5. STS Activates Ferroptosis-Triggered Low Expression of AMPK/NRF2 and CLOCK/BMAL1 in HUVECs

**Figure 5:**
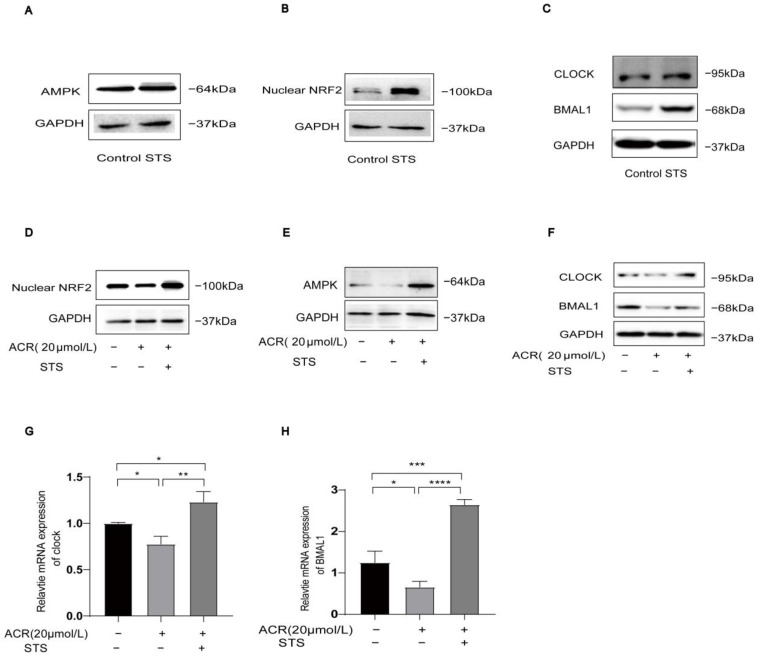
STS reverses acrolein-induced AMPK/NRF2 and CLOCK/BMAL1 downregulation in HUVECs. (**A**–**C**) The protein expression levels of AMPK*α*1/NRF2/CLOCK/BMAL1 were upregulated by using STS models. (**D**–**F**) The protein expression levels of AMPK*α*1/NRF2/CLOCK/BMAL1 were investigated following combined treatment with STS and acrolein. (**G**,**H**) The protein expression levels of AMPK*α*1/NRF2/CLOCK/BMAL1 were investigated following combined treatment with STS and acrolein. Data are expressed as mean ± standard deviation of at least three experiments and analyzed by one-way ANOVA, followed by the Bonferroni multiple comparison test. ns: no significance, * *p* < 0.05, ** *p* < 0.01, *** *p* < 0.001, and **** *p* < 0.0001.

To further explore the potential of IF in reversing low expression of AMPK/NRF2 and CLOCK/BMAL1 triggered by ferroptosis in cells, we used STS as a cell model in simulating IF. We investigated whether STS could activate the acrolein-induced low expression of AMPK/NRF2 and CLOCK/BMAL1. We treated cells in four groups: control, acrolein, STS, and acrolein + STS. Notably, protein expression levels of AMPK/NRF2 and CLOCK/BMAL1 were upregulated in the STS group compared to the control group (Figure 5A–C), while the acrolein + STS group reversed the inhibitory effect of the acrolein group on AMPK/NRF2 and CLOCK/BMAL1 (Figure 5D–F). The mRNA expression of CLOCK/BMAL1 was also upregulated in the acrolein + STS group compared to the acrolein group. In conclusion, STS effectively activates the acrolein-induced low expression of AMPK/NRF2 and CLOCK/BMAL1. (For all protein quantification data presented in Figure 5, please refer to Figure S4).

### 3.6. STS Reverses the Occurrence of Acrolein-Induced Ferroptosis and Lipid Peroxidation in HUVECs

**Figure 6:**
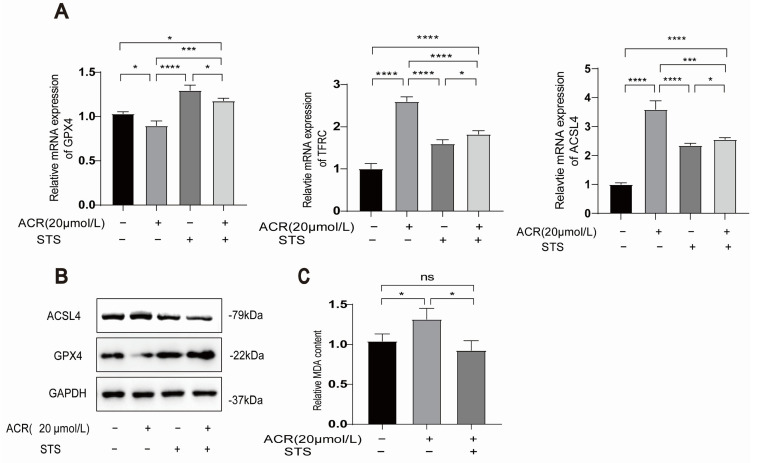
STS reverses acrolein-induced ferroptosis and lipid peroxidation. (**A**,**B**) The mRNA and protein expression levels of GPX4, TFRC, and ACSL4 were assessed following combined treatment with STS and acrolein. (**C**) Combined treatment with STS and acrolein was employed to determine the MDA content. Data are presented as mean ± standard deviation from at least three independent experiments and analyzed with one-way ANOVA, followed by the Bonferroni multiple comparison test. ns: no significance, * *p* < 0.05, *** *p* < 0.001, and **** *p* < 0.0001.

To investigate the potential of IF in reversing ferroptosis in cells, we employed an STS model in HUVECs. The HUVECs were divided into three groups: control, acrolein, and acrolein + STS. We observed an upregulation of the protein and mRNA levels of the ferroptosis marker GPX4 in the acrolein + STS group, while TFRC and ACSL4 were downregulated when compared to the acrolein group (Figure 6A,B). Additionally, lipid peroxide accumulation was identified as a key driver of ferroptosis, contributing to oxidative stress and cell death. The measurement of MDA content demonstrated lower levels in the acrolein + STS group compared to the acrolein group (Figure 6C). These results suggest that STS reverses both the occurrence of ferroptosis markers and lipid peroxidation triggered by acrolein in HUVECs. However, it is worth noting that while the STS group exhibited a significant reversal of ferroptosis markers, we did not conduct experiments combining STS with ferroptosis inhibitors such as ferrostatin-1. As such, we are unable to definitively attribute the protective effect of STS specifically to ferroptosis inhibition. Further and more in-depth research is still needed to elucidate this aspect. (For all protein quantification data presented in Figure 6, please refer to Figure S5).

## 4. Discussion

In this study, we discovered that IF reverses acrolein-induced ferroptosis and the downregulation of CLOCK in mice. Consistent with in vivo findings, we demonstrated that acrolein induces ferroptosis, leading to cell death, while STS reverses this effect. STS also activates the AMPK/NRF2-CLOCK/BMAL1 pathway, which is downregulated by acrolein, and reduces lipid peroxidation, thereby inhibiting ferroptosis. Furthermore, the AMPK/NRF2 and CLOCK/BMAL1 are mutually regulated. These observations provide novel insights into the mechanisms linking ferroptosis and the circadian clock and offer new theoretical evidence supporting the role of IF in the prevention and treatment of diseases associated with environmental pollution.

Environmental pollution has a considerable impact on human health [41]. Acrolein triggers and accelerates ferroptosis through the depletion of GPX4, upregulation of TFRC and ACSL4, and accumulation of lipid peroxides [42], which stimulates the production of reactive oxygen species (ROS) [43]. Acrolein-induced ferroptosis can trigger a host of adverse effects. For instance, it has been proposed that acrolein-induced ferroptosis impairs peripheral neurogenesis in zebrafish [44]. Additionally, other studies have demonstrated that acrolein can induce ferroptosis in pancreatic β-cells, resulting in insulin deficiency and resistance, which ultimately contribute to the development of diabetes [34]. We found that acrolein-induced ferroptosis can be reversed by ferrostatin-1, a known ferroptosis inhibitor. Additionally, we determined that acrolein inhibits the CLOCK/BMAL1-AMPK-NRF2 pathway, thereby contributing to the acceleration of ferroptosis.

Furthermore, because lipid peroxidation is one of the hallmarks of ferroptosis, it can contribute to oxidative stress reactions in vascular endothelial cells, which subsequently leads to vascular endothelial dysfunction [45]. Therefore, ferroptosis is considered a critical early event in the development of atherosclerosis [46]. Our previous study has indicated that IF improves acrolein-induced atherosclerosis by regulating the expression of CLOCK/BMAL1 [11]. Therefore, we explored whether IF has a reversing effect on ferroptosis through the circadian clock by in vivo and in vitro experiments. The results showed that the downregulation of GPX4 and upregulation of TFRC and ACSL4 induced by ferroptosis were reversed by IF in vivo. Consistent with the in vivo findings, STS also reversed ferroptosis and lipid peroxidation in in vitro experiments using HUVECs.

Scientific evidence has demonstrated that IF can reduce both lipid peroxidation and mitochondrial dysfunction, thereby alleviating the characteristic cellular and mouse damage induced by ferroptosis [26]. In this study, we explored whether IF could inhibit ferroptosis through the circadian clock. Through in vivo experiments using an APOE-/- mice model of ferroptosis, we found that IF inhibited both the occurrence and progression of ferroptosis, accompanied by an increase in the expression of CLOCK/BMAL1 that were downregulated as a result of ferroptosis. Consistent with the in vivo findings, STS also reversed the occurrence of ferroptosis and the low expression of CLOCK/BMAL1 genes induced in HUVECs. Due to the relationship between IF and CLOCK/BMAL1, we hypothesized that CLOCK/BMAL1 may also have a regulatory effect on ferroptosis. Consistent with this hypothesis, CLOCK/BMAL1 can regulate the development of ferroptosis in HUVECs. When CLOCK/BMAL1 were knocked down, the ferroptosis marker GPX4 was downregulated, while TFRC and ACSL4 were upregulated. Conversely, when CLOCK was overexpressed, GPX4 was upregulated, and both TFRC and ACSL4 were downregulated. To our knowledge, this is the first report indicating that IF can serve as an intervention to treat acrolein-induced ferroptosis via the circadian clock. While this research has elucidated the pivotal role of CLOCK/BMAL1 in inhibiting ferroptosis and explored how CLOCK/BMAL1 is regulated by acrolein and IF, further in-depth investigation is still needed to uncover the underlying mechanisms.

In addition to CLOCK/BMAL1, IF is closely associated with energy-responsive molecules, such as AMPK. As an energy regulator, AMPK plays a crucial role in the dysregulation of diabetes, cardiometabolic diseases, and cancer [47]. Numerous recent studies have highlighted the pivotal role of AMPK activation in ameliorating various inflammatory [48], ischemia/reperfusion [49], and oxidative stress-related diseases [50]. Cells with elevated basal AMPK activity exhibit resistance to ferroptosis, whereas the inactivation of AMPK renders these cells susceptible to ferroptosis [51]. Previous investigations have confirmed that AMPK activation can inhibit lipid metabolism disorder-induced ferroptosis in macrophages by upregulating GPX4 expression [52]. NRF2 serves as a transcription factor involved in the stress response and various physiological reactions [53]. Due to its ability to target numerous ferroptosis-related genes, NRF2 has been recognized as a key regulator of ferroptosis, occupying a pivotal position in this process. In addition, the activation of NRF2 in preventing ferroptosis and related pathogenesis is mediated by AMPK. It is noteworthy that the observed overlapping cellular responses upon activation of AMPK or NRF2, as well as the common stressors that impinge on both AMPK and NRF2 signaling pathways, provide a basis for the hypothesis that these two signaling pathways may be interdependent and act in concert to restore cellular homeostasis [38]. Therefore, activation of the AMPK/NRF2 pathway can effectively regulate ferroptosis [54,55]. In this study, we explored the role of the AMPK/NRF2 pathway in regulating ferroptosis. However, it should be noted that the current study is limited by the absence of NRF2 siRNA experiments. This limitation underscores the necessity for further investigation in future research. In addition, we also demonstrated that IF acts as an effective mode of AMPK/NRF2 activation, attenuating lipid peroxidation and subsequent ferroptosis.

Given that STS reversed the occurrence of ferroptosis and induced the low expression of CLOCK/BMAL1 and AMPK/NRF2, we further investigated whether AMPK/NRF2 and CLOCK/BMAL1 have mutual regulation. Our results demonstrated that AMPK/NRF2 and CLOCK/BMAL1 positively regulate each other.

Additionally, it is worth noting that IF triggers autophagy—the body’s cellular cleanup process—by lowering insulin and mTOR activity while activating AMPK and ketosis [56,57]. This typically peaks after 16–48 h of fasting. As a result, it enhances cellular repair, reduces inflammation, and may protect against aging, neurodegeneration, and metabolic diseases [58]. This phenomenon warrants further investigation in conjunction with the findings presented in this research.

In summary, our study revealed that IF exerts inhibitory effects on ferroptosis and lipid peroxidation. Additionally, IF attenuates the cell death and downregulation of CLOCK/BMAL1-AMPK/NRF2 induced by ferroptosis. Additionally, we identified a regulatory role of CLOCK/BMAL1 in ferroptosis. Furthermore, we discovered a reciprocal regulation between CLOCK/BMAL1 and AMPK/NRF2. Upon validation by further research, the AMPK-NRF2/CLOCK-BMAL1 regulatory axis could represent a novel research target for investigating the mechanisms of ferroptosis and circadian clock interactions in the future. This could provide new theoretical evidence for the role of IF in the prevention and treatment of diseases related to environmental pollution.

## 5. Conclusions

In conclusion, our findings suggest that IF modulates the CLOCK/BMAL1-AMPK/NRF2 pathway to alleviate acrolein-induced ferroptosis, offering a potential strategy to address health issues related to environmental pollution.

## Figures and Tables

**Table 1:** The primers for qPCR (human).

Primer	Forward Primer (5′-3′)	Reverse Primer (5′-3′)
GPX4	ACAAGAACGGCTGCGTGGTGAA	GAGCTAGAAATAGTGGGGCAGGT
ACSL4	TGGCAAAGGAGCAGATTAGTAGG	TCACTTAGGATTTCCCTGGTCC
TFRC	ACCATTGTCATATACCCGGTTCA	CAATAGCCCAAGTAGCCAATCAT
AMPK	GTAGTAAAAACAGGCTCCACGAA	CACCAGAAAGGATCTGTTGGA
NRF2	ACAAGAACGGCTGCGTGGTGAA	GAGCTAGAAATAGTGGGGCAGGT
CLOCK	ATGGTTTTTACCGTAAGCTGTAG	CTCGCGTTACCAGGAAGCAT
BMAL1	AGGCCCACAGTCAGATTGAAA	CCAAAGAAGCCAATTCATCAATG
GAPDH	GGAGCGAGATCCCTCCAAAAT	GGCTGTTGTCATACTTCTCATGG

**Table 2:** The primers for qPCR (mice).

Primer	Forward Primer (5′-3′)	Reverse Primer (5′-3′)
CLOCK	CCTATCCTACCTTGGCCACACA	TCCCGTGGAGCAACCTAGAT
GPX4	GCAACCAGTTTGGGAGGCAGGAG	CCTCCATGGGACCATAGCGCTTC
ACSL4	TGAACGTATCCCTGGACTAGG	TCAGACAGTGTAAGGGGTGAA
TFRC	GTGGAGTATCACTTCCTGTCGC	CCCCAGATATGTCGGAAAGG
GAPDH	TGGCCTTCCGTGTTCCTA	GAGTTGCTGTTGAAGTCGCA

**Table 3:** Target sequence of siRNA for transfection.

Gene	Sequence
siCLOCK-01	CAAAGAAGGTCATCATTTA
siCLOCK-02	GGAGCCATCTACCTATGAA
siCLOCK-03	GCTTCCTGGTAATGCTAGA

## Data Availability

The sources of the data used for this research are provided.

## Supplementary Materials

The following supporting information can be downloaded at: https://www.mdpi.com/article/10.3390/toxics13050369/s1, Figure S1: Quantification of cellular protein levels corresponding to Figure 2; Figure S2: Quantification of cellular protein levels corresponding to Figure 3; Figure S3: Quantification of cellular protein levels corresponding to Figure 4; Figure S4: Quantification of cellular protein levels corresponding to Figure 5; Figure S5: Quantification of cellular protein levels corresponding to Figure 6.
