# Supplementary material for: Acrolein-Triggered Ferroptosis and Protection by Intermittent Fasting via the AMPK/NRF2-CLOCK/BMAL1 Pathway
Source: Toxics. 2025 May 1;13(5):369. doi: 10.3390/toxics13050369 (PMC12115751; doi:10.3390/toxics13050369)
Supplement: Supplementary file 1 [file toxics-13-00369-s001.zip › toxics-3574140-supplementary.pdf]

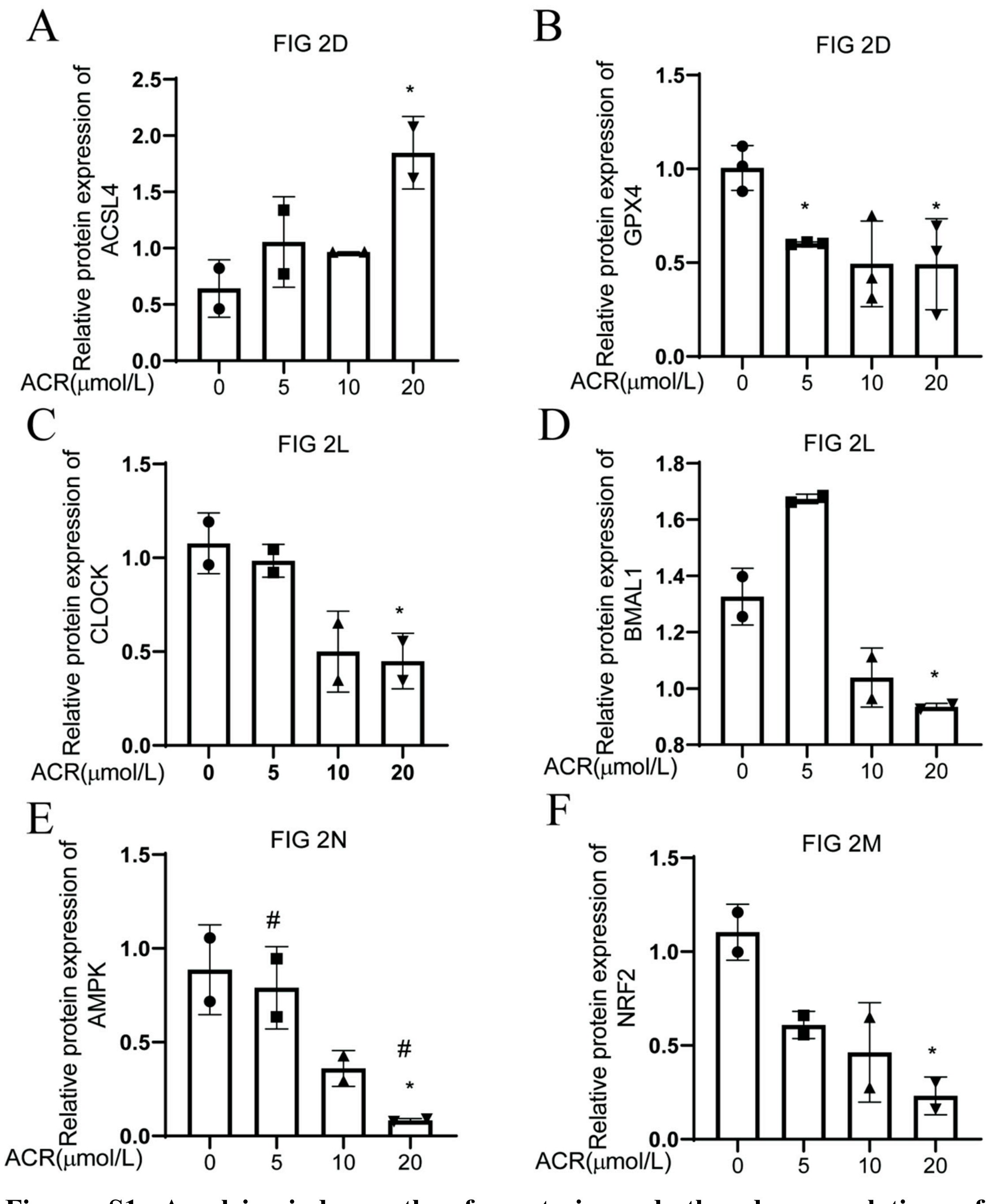

Figure S1 Acrolein induces the ferroptosis and the downregulation of AMPK/NRF2 and CLOCK/BMAL1 in HUVEC cells.

(A-F) Protein expression levels of GPX4, ACSL4, CLOCK, BMAL1, AMPK, NRF2 in HUVEC were assessed after treatment with various concentrations of acrolein. (*P < 0.05, **P < 0.01, ***P < 0.001, and ****P < 0.0001)

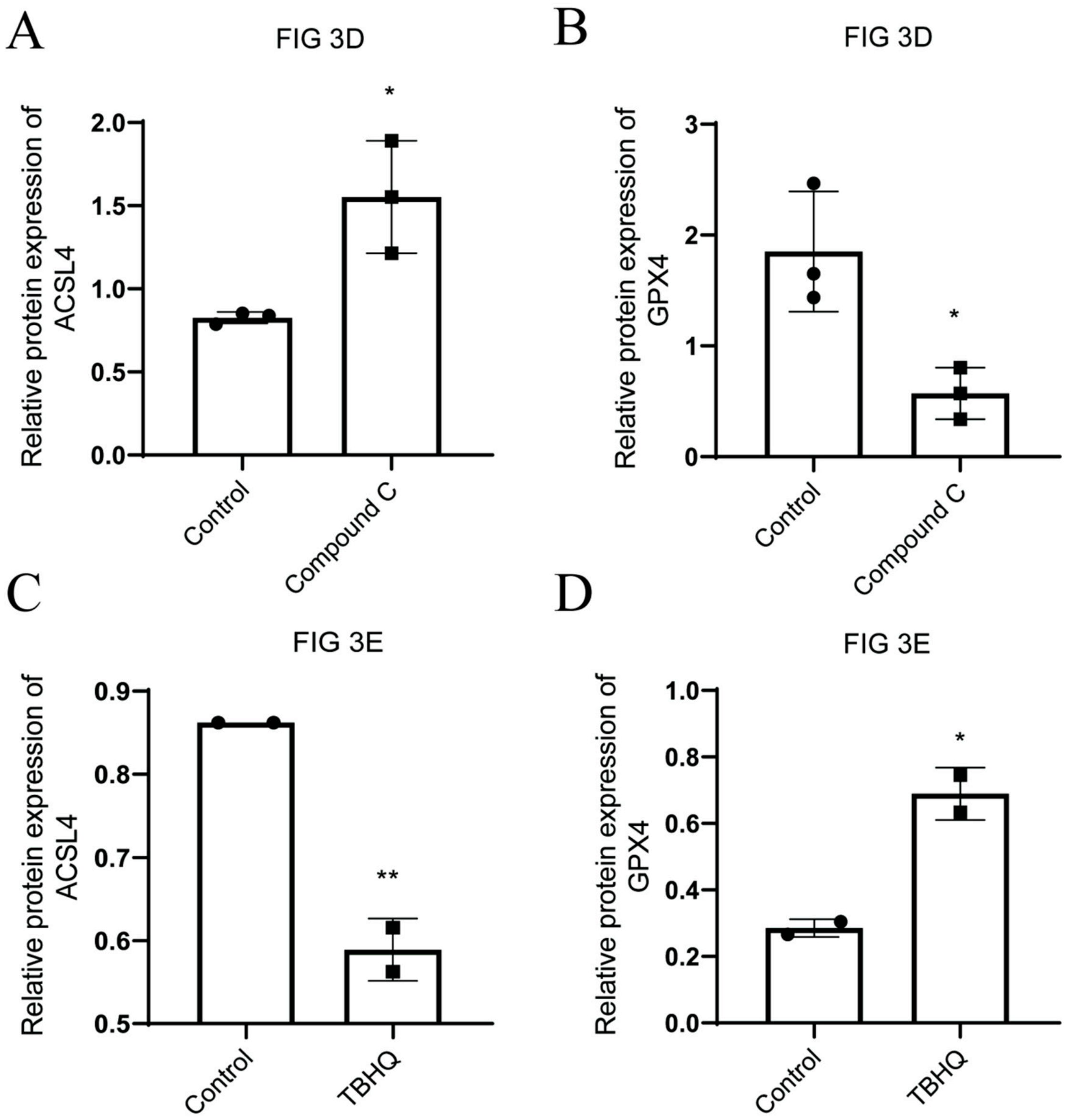

Figure S2 CLOCK/BMAL1 and AMPK/NRF2 regulates ferroptosis in HUVEC cells.

(A-B) Protein expression levels of ACSL4 and GPX4 in HUVEC were assessed after treatment with AMPK inhibitor (Compound C). (C-D) Protein expression levels of ACSL4 and GPX4 in HUVEC were assessed after treatment with NRF2 activator (TBHQ). (*$P < 0.05$, **$P < 0.01$, ***$P < 0.001$, and ****$P < 0.0001$)

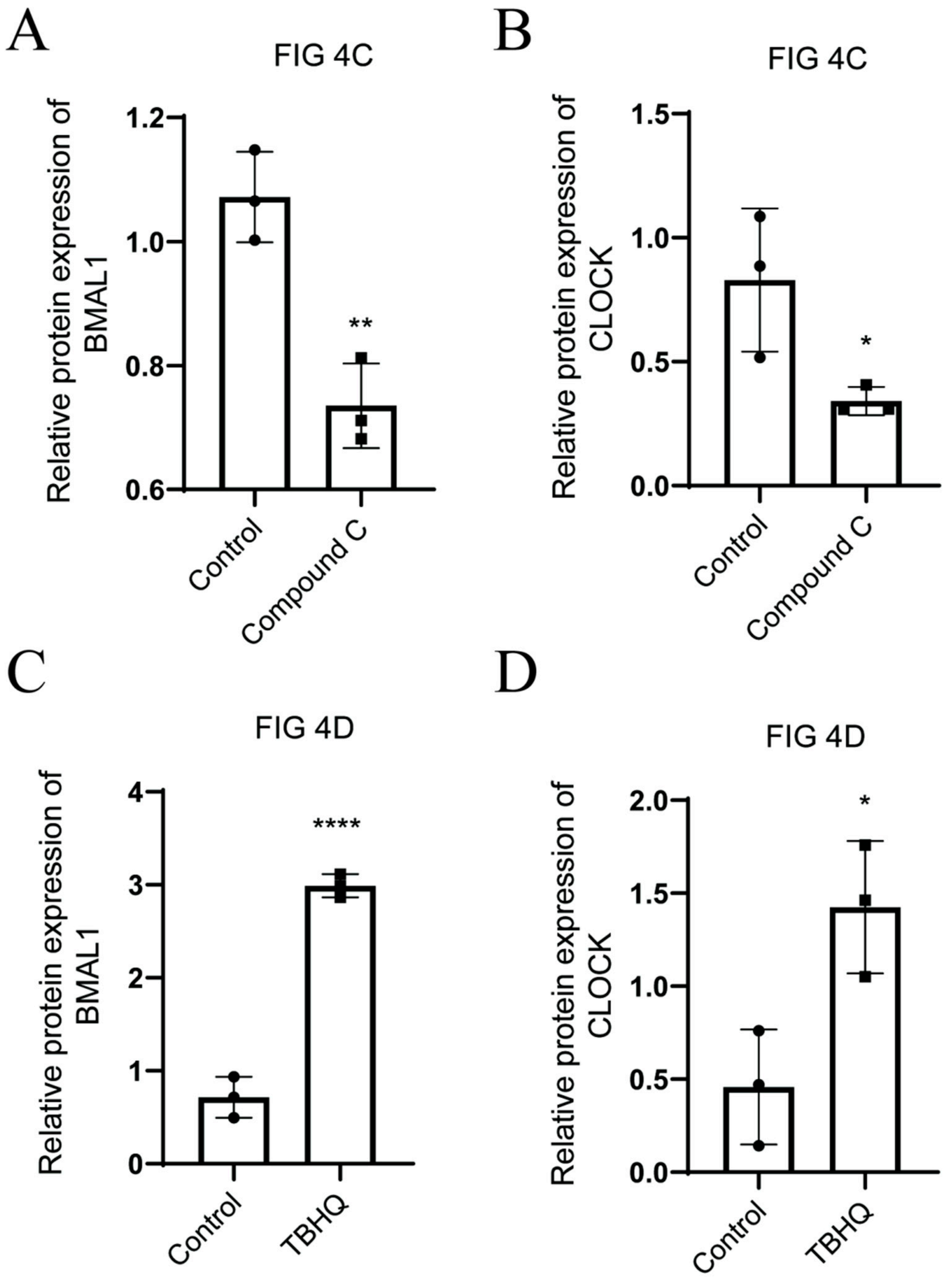

Figure S3 CLOCK/BMAL1/AMPK/NRF2 expression and mutual regulation in HUVEC cells.

(A-B) Protein expression levels of CLOCK and BMAL1 in HUVEC were assessed after treatment with AMPK inhibitor (Compound C). (C-D) Protein expression levels of CLOCK and BMAL1 in HUVEC were assessed after treatment with NRF2 activator (TBHQ). (*$P < 0.05$, **$P < 0.01$, ***$P < 0.001$, and ****$P < 0.0001$)

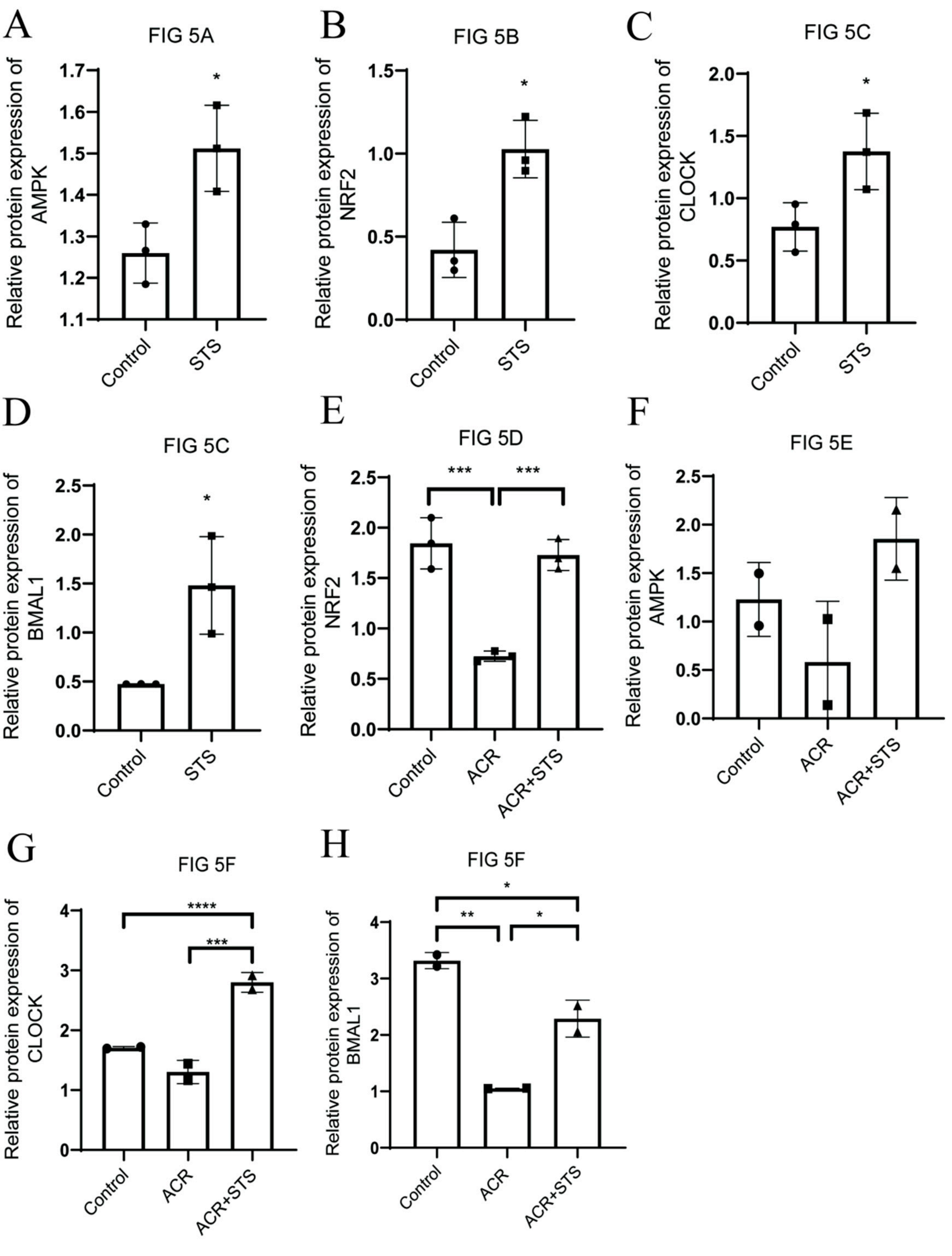

Figure S4 STS reverses acrolein-induced AMPK/NRF2 and CLOCK/BMAL1 downregulation in HUVEC cells.

(A-D) The protein expression levels of AMPK/NRF2/CLOCK/BMAL1 were assessed after treatment with STS models. (E-H) The protein expression levels of AMPK/NRF2/CLOCK/BMAL1 were assessed after combined treatment with STS and acrolein. (*P < 0.05, **P < 0.01, ***P < 0.001, and ****P < 0.0001)

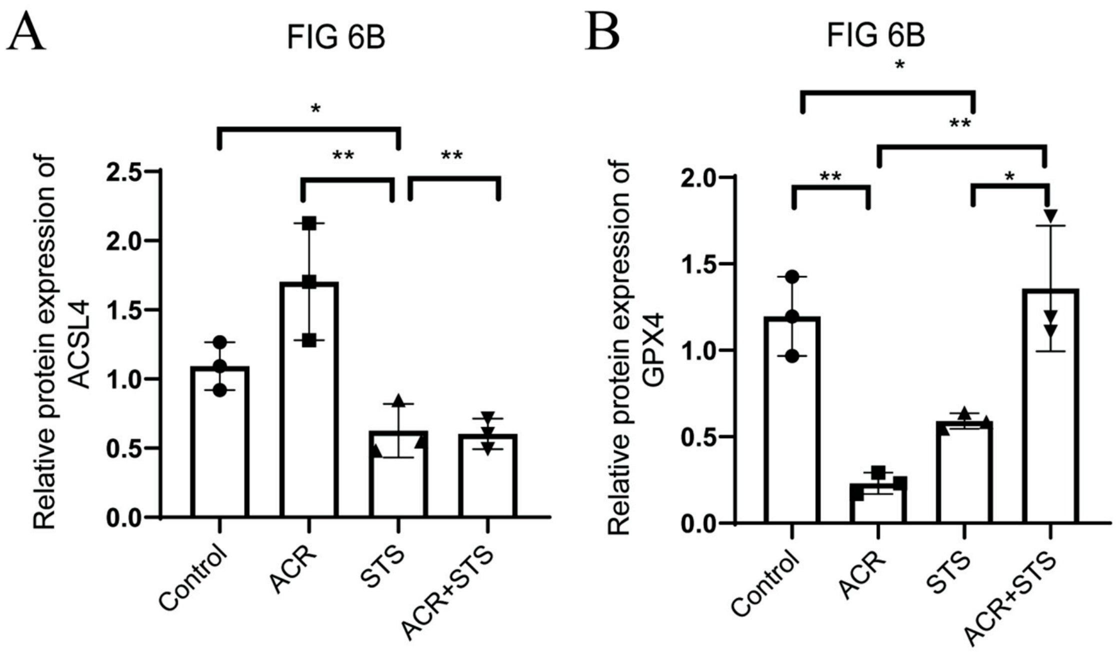

Figure S5 STS reverses acrolein-induced ferroptosis and lipid peroxidation.

(A-B) The protein expression levels of GPX4 and ACSL4 were assessed following combined treatment with STS and acrolein. (*P < 0.05, **P < 0.01, ***P < 0.001, and ****P < 0.0001)
